# Microstructure and Mechanical Properties of Novel Lightweight TaNbVTi-Based Refractory High Entropy Alloys

**DOI:** 10.3390/ma15010355

**Published:** 2022-01-04

**Authors:** Ao Fu, Yuankui Cao, Yuxi Liu, Shenghang Xu

**Affiliations:** 1 State Key Laboratory of Powder Metallurgy, Central South University, Changsha 410083, China; iceiceice_isu@hotmail.com (A.F.); yuxiliu@csu.edu.cn (Y.L.); 2 Institute of Laser Advanced Manufacturing, Zhejiang University of Technology, Hangzhou 310023, China; 3 College of Materials Science and Engineering, Zhejiang University of Technology, Hangzhou 310014, China; shenghangxu@zjut.edu.cn

**Keywords:** refractory high entropy alloy, microstructure, powder metallurgy, sintering, mechanical property

## Abstract

A series of novel lightweight TaNbVTi-based refractory high entropy alloys (RHEA) were fabricated through ball-milling and spark plasma sintering (SPS). The reinforced phase of TiO precipitates were in-situ formed due to the introduction of Al_2_O_3_ ceramic particles. The RHEA with 15% Al_2_O_3_ exhibits a high compressive yield strength (1837 MPa) and a low density (7.75 g/cm^3^) with an adequate ductility retention. The yield strength and density are 32% higher and 15% lower, respectively, compared to the RHEA without Al_2_O_3_ addition. The specific yield strength (237 MPa cm^3^/g) of the RHEAs is much higher than that of other reported RHEAs, and is mainly ascribed to the introduction of high volume fraction of Al_2_O_3_ additives, resulting in solid solution strengthening and precipitation strengthening. Meanwhile, the ductile matrix is responsible for the good compressive plasticity.

## 1. Introduction

High entropy alloys (HEAs) are generally consisted of four or more principal elements in either equi-atomic or near equi-atomic composition, and tend to form simple solid solution structures, e.g., face-centered cubic (fcc), body-centered cubic (bcc) and or hexagonal close-packed (hcp), instead of complex phases or intermetallic compounds [1,2]. HEAs have recently received much attention owing to their unprecedented promising properties, such as outstanding strength, excellent fracture toughness, remarkable wear and corrosion resistance, etc. [2,3,4,5,6]. This concept provides enormous capabilities for the development of novel alloys for application in extreme environments [3,7]. Recently, refractory high entropy alloys (RHEAs) consisted of refractory elements, such as W, Mo, Ta, Nb, V, Ti, Zr, etc., have attracted increasing attention by virtue of their superior mechanical properties at room and elevated temperature [8,9,10,11]. Unfortunately, their high density (up to 10–14 g/cm^3^) and consequent low specific strength restrict their engineering application.

Adding lightweight elements into RHEAs can effectively reduce the density and improve the specific strength [12,13,14,15]. For example, Senkov et al. fabricated Al_0.4_Hf_0.6_NbTaTiZr RHEA by adding Al into the HfNbTaTiZr RHEA. The density of the alloy decreased from 9.94 to 9.05 g/cm^3^, and the specific yield strength increased from 93.56 to 203.43 MPa∙cm^3^/g [16]. However, their plasticity deteriorated significantly. Recently, Yang et al. found that NbTiVTaAl_0.25_ RHEA with Al addition still have outstanding compressive plasticity greater than 50% [17]. Nevertheless, the specific strength of this alloy is only 151.48 MPa∙cm^3^/g, which has the potential for further improvement. In this work, a series of lightweight TaNbVTi-based RHEAs were fabricated by mechanical alloying (MA) followed by spark plasma sintering (SPS). The microstructural evolution and its effects on mechanical properties were analyzed.

## 2. Experimental Procedures

**Figure 1:**
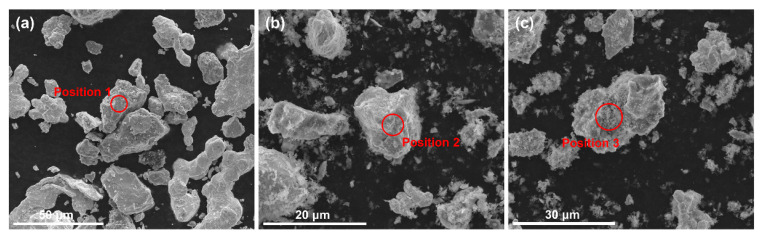
Morphology of the as-milled powders. (**a**) TaNbVTi-0, (**b**) TaNbVTi-1 and (**c**) TaNbVTi-2 powders.

The TaNbVTi-based RHEAs were prepared by powder metallurgy (P/M) method. High purity (>99.5 wt.%) Ta, Nb, V, Ti elemental powders in equiatomic composition were mixed with Al_2_O_3_ powders as the raw materials, and then blended with a planetary ball-millers for 6 h at 120 rpm under argon atmosphere at a temperature of 25 °C. The average particle size of Ta, Nb, V, Ti and Al_2_O_3_ powders is 24.5 μm, 26.1 μm, 22.6 μm, 29.1 μm and 0.3 μm, respectively. The content of interstitial impurities of these powders are listed in Table 1. In this work, three volume fractions of Al_2_O_3_ powders (0%, 10% and 15%) were added to in-situ generate particles reinforced RHEAs, namely, TaNbVTi-0, TaNbVTi-1 and TaNbVTi-2, respectively. Figure 1 shows the morphology of the as-milled powders. The average particle size and chemical composition of these powders are given in Table 2. It can be seen that all these powders are in irregular shape. The content of Al and O in the as-milled powders increases with the increase of Al_2_O_3_ powders. Finally, the as-milled powders were consolidated via SPS machine (FCT HP D 25/3,Frankenblick, German) at 1700 °C with a heating rate of 100 °C/min. The sintering process was held for 10 min with a constant pressure of 30 MPa. The dimension of the sintered bulks is 10 mm in height and 40 mm in diameter.

The oxygen content was determined by the fusion method on a Leco O/N analyzer. The composition was analyzed by chemical methods. The particle size distribution was investigated by a laser particle size analyzer (MICRO-PLUS). The phase analysis was performed by an X-ray diffraction analyses (XRD, Advance D8, Billerica, MA, USA) with Cu Ka radiation. The XRD analyses were conducted at a 2θ from 10–80° with a scan rate of 5°/min. The microstructure was characterized by a scanning electron microscope (SEM, Helios Nanolab 600i, Hillsboro, OR, USA) equipped with a backscatter electron (BSE) detector and an energy dispersive X-ray spectroscopy (EDX) device. The elemental distribution was analyzed by an electron probe microanalysis (EPMA, JXA 8530F, Tokyo, Japan). The density of the specimens was measured by Archimedes method. Cubic specimens with a dimension of 8 mm × 8 mm × 8 mm for density test were obtained from the center of the sintered bulks. Three samples were tested for the same composition, and the average value was adopted. Cylindrical specimens (Φ 6 mm × 9 mm) for compressive test were cut from the sintered bulks by using an electrical discharge machining. Before testing, the surface of test specimens was polished by a 2000-grit SiC paper to eliminate scratches. Room temperature compressive test was carried out on a universal testing machine (Instron-3369, Norwood, MA, USA) at a strain rate of 1 × 10^−3^ s^−1^. All the specimens were compressed until fracture. Tensile tests were carried out on an MTS Landmark test machine at a strain rate of 1 × 10^−3^ s^−1^ by using “I” sharp samples with a gauge length of 10 mm and a rectangular cross-section of 2 mm × 2 mm.

## 3. Results and Discussion

**Figure 2:**
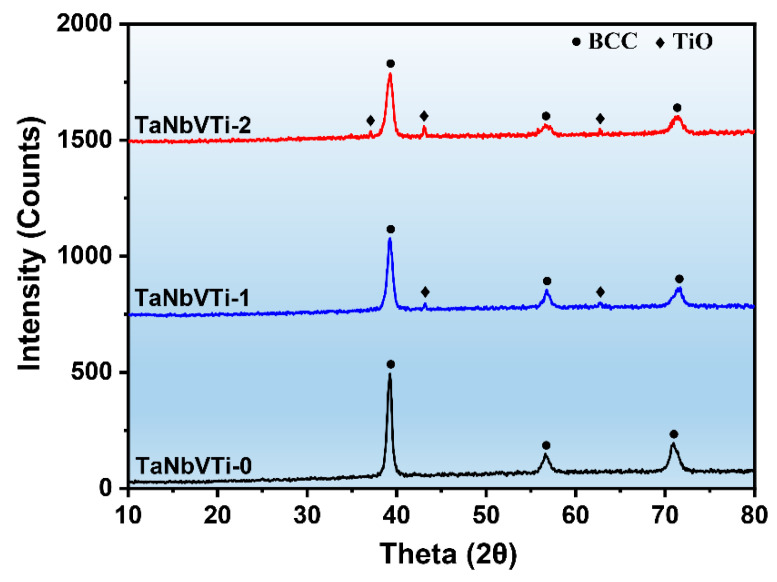
XRD patterns of the sintered TaNbVTi-0, TaNbVTi-1 and TaNbVTi-2 RHEAs.

Figure 2 shows the XRD pattern of the sintered bulks. It is evident that the TaNbVTi-0 RHEA exhibits a single-phase BCC structure, and the related lattice parameter is determined to be ~0.323 nm. After the addition of Al_2_O_3_, some extra diffraction peaks are detected in the TaNbVTi-1 RHEA, and the intensity increased as the increase of Al_2_O_3_ addition, indicating more second phase forms in the TaNbVTi-2 RHEA. According to the JCPDS cards (No. 72-0020), the second phase can be identified as TiO phase, implying the formation of TiO phase in the sintered bulks, which is consistent with the results reported by Xin et al. [18].

**Figure 3:**
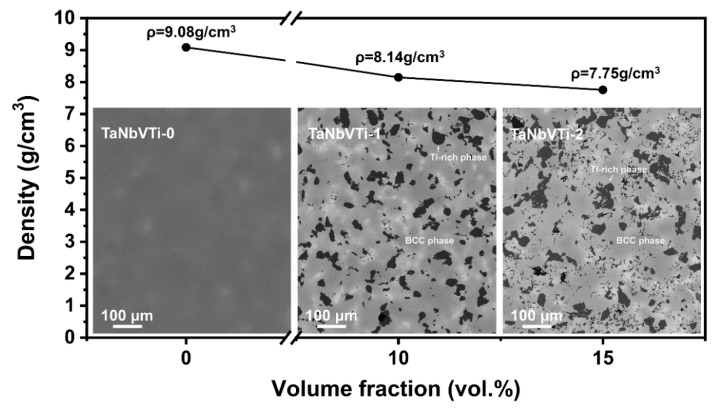
Densities and SEM images of the sintered TaNbVTi-0, TaNbVTi-1 and TaNbVTi-2 RHEAs.

**Figure 4:**
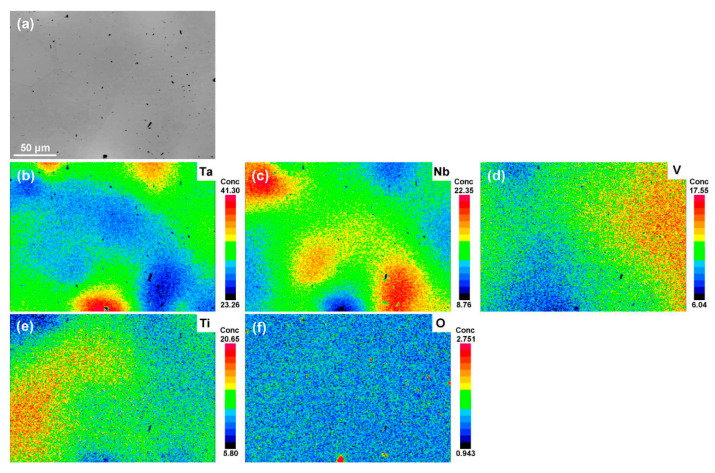
EPMA results of the sintered TaNbVTi-0 RHEA: (**a**) SEM images; (**b**–**f**) distributions of element Ta, Nb, V, Ti and O.

**Figure 5:**
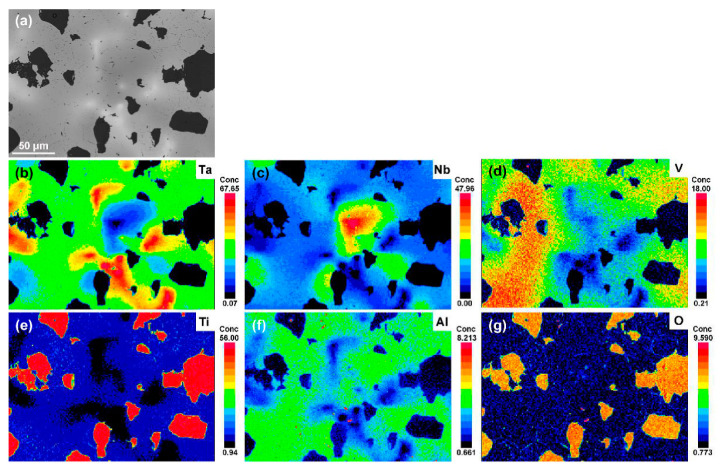
EPMA results of the sintered TaNbVTi-2 RHEA: (**a**) SEM images; (**b**–**g**) distributions of element Ta, Nb, V, Ti, Al and O.

Figure 3 shows the density and microstructural evolution of the as-sintered bulks. It can be seen that the density of the bulks decreases rapidly from 9.08 g/cm^3^ of TaNbVTi-0 RHEA to 7.75 g/cm^3^ of TaNbVTi-2 RHEA with the incremental Al_2_O_3_ addition. The embedded images show the microstructural characteristic of the TaNbVTi-0, TaNbVTi-1 and TaNbVTi-2 RHEAs. After sintering, the bulks can be consolidated to nearly full dense, and there are few residual pores or shrinkage defects in the microstructure. Slight component segregation can be observed in the TaNbVTi-0 RHEA, and the microstructure is composed of two distinguishable regions, bright regions and dark regions, indicating an incomplete alloying process. Figure 4 shows the EPMA mapping of the TaNbVTi-0 RHEA. The bright regions are enriched in Ta while the dark regions are enriched in Ta, Nb and V, which is consistent with the results reported by Guo et al. [8]. After addition of 10 vol.% Al_2_O_3_, a large number of particles can be clearly found in the TaNbVTi-1 RHEA (Figure 3). The volume fraction of particles increases significantly with more addition of Al_2_O_3_ in TaNbVTi-2 RHEA, which is consist with previous XRD results. As shown in Figure 3, the microstructure of the TaNbVTi-1 and TaNbVTi-2 RHEAs exhibits two distinguishable regions: one is dark matrix, and the other is black particle. The details of the elemental distribution of the two regions in the TaNbVTi-2 RHEA are investigated by EPMA mapping (Figure 5). According to the results, the black regions with distinct boundaries are enrich in Ti and O. It has been widely reported that Al and O elements are introduced from the initial Al_2_O_3_ powders during the MA process [19,20,21]. Then the Ti in the matrix is favorable to react with O to form TiO particles when the sintering temperature is higher than 1057 °C [18], which leads to the decrease of Ti content in the matrix significantly. The TiO particles can hinder the movement of dislocations, leading to the strength enhancement by precipitation strengthening effect [22,23]. Meanwhile, Al atoms decomposed from Al_2_O_3_ diffuse and dissolve into the matrix, and so that the dark regions are enriched in Ta, Nb, V, Ti and Al, but depleted in O. Since misfit volume of Al is large, a small amount of interstitial Al atoms will cause remarkable solid solution strengthening effect, and thereby resulting in strength improvement [24].

**Figure 6:**
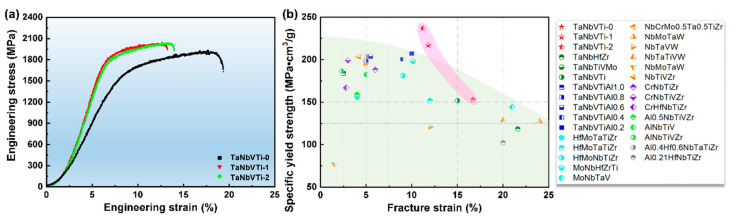
(**a**) Typical compression engineering stress-strain curves of the sintered TaNbVTi-0, TaNbVTi-1 and TaNbVTi-2 RHEAs; (**b**) comparison of compressive specific yield strength at room temperature for the current TaNbVTi-based RHEAs and typical RHEAs [25,26].

The compressive curves of the TaNbVTi-0, TaNbVTi-1 and TaNbVTi-2 RHEAs are shown in Figure 6a. The yield strength, ultimate strength and the fracture strain of the TaNbVTi-0 RHEA are 1391 MPa, 1932 MPa and 16.7%, respectively. And the TaNbVTi-2 RHEA has enhanced yield strength of 1837 MPa and ultimate strength of 2030 MPa, but its fracture strain decreases to 11.2%. During the compression testing, no visible damage or macro-crack was observed before attaining the maximum load. Apparently, for the TaNbVTi-1 and the TaNbVTi-2 RHEAs, the introduction of incremental Al_2_O_3_ leads to slight sacrifice in plasticity, but the yield strength and specific yield strength improve significantly, mainly due to the remarkable solid solution strengthening effect caused by Al in the matrix and the precipitation strengthening effect caused by the TiO particles. At the same time, the TaNbVTi-2 RHEA exhibits the highest specific yield strength of 237 MPa cm^3^/g, which is slight higher than that of TaNbVTi-1 RHEA (217 MPa cm^3^/g). Figure 6b presents fracture strain dependence of compressive specific yield strength of the TaNbVTi-based RHEAs compared with other previously reported typical RHEAs [25,26]. Surprisingly, the results highlight the extraordinary mechanical properties of the TaNbTiV based RHEAs compared with many other previously reported RHEAs with moderate plasticity. These exceptional mechanical features suggest a promising method for enhancing RHEAs through the addition of ceramic reinforcements. In addition, the tensile properties of the RHEAs were also tested for comparison, as shown in Table 3. The tensile strength is close to compressive strength, while the tensile elongation is obviously lower than the compressive fracture strain, main due to the stress concentration induced by the TiO particles during tensile test.

## 4. Conclusions

(1) Novel lightweight TaNbVTi-based RHEAs has been successfully developed through the addition of Al_2_O_3_ ceramic particles. The introduction of Al_2_O_3_ can promotes the formation of TiO precipitates in the BCC matrix. Meanwhile, Al atoms decomposed from Al_2_O_3_ could diffuse and dissolve into the matrix.
(2) The TaNbVTi-2 RHEA has a relative low density of 7.75 g/cm^3^ with a high compressive yield strength of 1837 MPa, which are 32% higher and 15% lower, respectively, compared to the RHEA without Al_2_O_3_ addition. The specific yield strength of TaNbVTi-2 RHEA is better than most reported RHEAs. The newly developed RHEAs are promising for applying in aerospace field (such as aero-engine, nozzle, etc.) due to the high specific strength.
(3) The improved strength is mainly ascribed to the introduction high volume fraction of Al_2_O_3_ additives, resulting in solid solution strengthening and precipitation strengthening. Moreover, the ductile matrix is responsible for the good compressive plasticity.

## Figures and Tables

**Table 1:** Average particle size and content of elements C, H, O and N in the raw powders.

Raw Powders	Average Particle Size (μm)	C (wt.%)	O (wt.%)	H (wt.%)	N (wt.%)
Ta	24.5	0.020	0.130	0.0008	0.0064
Nb	26.1	0.011	0.340	0.0015	0.0220
V	22.6	0.028	0.290	0.0014	0.0096
Ti	29.1	0.018	0.280	0.0147	0.0150
Al_2_O_3_	0.3	0.013	16.56	0.0653	0.0070

**Table 2:** Average particle size and chemical composition of the as-milled TaNbVTi-0, TaNbVTi-1 and TaNbVTi-2 powders.

As-Milled Powders	Average Particle Size (μm)	Nb (at.%)	Ta (at.%)	Ti (at.%)	V (at.%)	Al (at.%)	O (at.%)
TaNbVTi-0	21.5	22.66	27.61	24.25	23.17	0	2.31
TaNbVTi-1	23.2	19.27	23.39	20.33	19.58	6.73	10.70
TaNbVTi-2	24.3	17.47	22.21	18.57	17.67	9.72	14.37

**Table 3:** Comparison in compression properties and tensile properties of the TaNbVTi-based RHEAs.

Alloys	Testing Method	Yield Strength (MPa)	Fracture Strength (MPa)	Fracture Strain/Elongation (%)
TaNbVTi-0	Compression	1391	1932	16..7
TaNbVTi-1		1776	2004	12.0
TaNbVTi-2		1837	2030	11.2
TaNbVTi-0	Tensile	1345	1724	7.2
TaNbVTi-1		1694	1908	2.3
TaNbVTi-2		1762	1927	2.1

## Data Availability

The data presented in this study are available on request from the corresponding author.
